# Linezolid-induced black hairy tongue in a patient treated for idiopathic granulomatous mastitis: a case report

**DOI:** 10.1097/MS9.0000000000000834

**Published:** 2023-05-10

**Authors:** Yashendra Sethi, Inderbir Padda, Matthew Fulton, Oroshay Kaiwan, Hitesh Chopra, Talha Bin Emran

**Affiliations:** a PearResearch; b Government Doon Medical College, Dehradun; c Chitkara College of Pharmacy, Chitkara University, Rajpura, Punjab, India; d Richmond University Medical Center, Staten Island, NY; e Universidad de Monterrey, San Pedro Garza García, Nuevo León, Mexico; f Northeast Ohio Medical University, OH; g Department of Pharmacy, BGC Trust University Bangladesh, Chittagong, Bangladesh; h Department of Pharmacy, Faculty of Allied Health Sciences, Daffodil International University, Dhaka, Bangladesh

**Keywords:** Adverse event, antimicrobialbht, black hairy tongue, drug-induced, linezolid

## Abstract

Linezolid-induced black hairy tongue (BHT) is a highly scarce adverse event of linezolid therapy. To date, there are very few reported cases in contemporary literature. The onset and mechanism of BHT are also not well understood.

**Case report:**

In this article, the authors report a case of BHT in a 28-year-old female following 5 days of therapy with linezolid. The patient recovered well within few days of discontinuation of the drug and maintaining oral hygeine. Patient reassurance and counselling was integral to the management.

**Discussion and conclusion:**

This case report and review depict a rare adverse effect of linezolid and discuss its clinical implications aiding healthcare professionals in an early diagnosis and cromulent management strategy. The authors also present a compilation of previously reported literature on linezolid-induced BHT to support the discussion.

## Introduction

Highlights

- Linezolid-induced black hairy tongue is a rare seen adverse effect.
- Black hairy tongue presents as hypertrophy and discoloration of the filiform papillae of the tongue.
- The discoloration can be black, brown, green or blue.
- Management involves patient reassurance and counseling, maintaining oral hygiene and may require reduction in dose or discontinuation of the drug (Linezolid).

Linezolid, an antimicrobial belonging to the oxazolidinone group, is a potent antibiotic active against a catena of Gram-positive bacteria, including methicillin-resistant *Staphylococcus aureus* and Vancomycin-resistant *Enterococci* ^1^. The drug is widely used for resistant chronic infections of the lungs, nosocomial pneumonia, community-acquired pneumonia, breast infections and skin infections. Linezolid therapy has become widely popular over the last decade for use in tough-to-counter bacterial infections. The mechanism of action includes inhibition of bacterial protein synthesis, with the drug binding on bacterial 23S ribosomal RNA of 50S subunit, preventing the formation of functional 70S initiation complex, hence preventing the translation^2^. The drug has near 100% oral bioavailability and is usually well tolerated. Common side-effects include nausea, vomiting, headache, diarrhoea, and rarely bone marrow suppression, and peripheral neuropathy with prolonged use^3^. Black hairy tongue (BHT) is a very rare side-effect of linezolid which presents as hypertrophy and discoloration of the filiform papillae of the tongue^4^. The colour of discoloration can range from black to brown, green, or blue^5^. The reported incidence of BHT in phase III of linezolid clinical trials was only 0.2%^6,7^. We report a unique case of linezolid-induced BHT in a young patient treated for idiopathic granulomatous mastitis. This case report has been reported in line with the Surgical CAse REport (SCARE) Guidelines^8^.

## Methodology

The authors of this article report a rare adverse event of linezolid therapy. Informed consent was taken from the patient to report the data and images. A literature review was also performed using the electronic databases PubMed, Pubmed Central (PMC), and Google Scholar. Pertinent data were collected and reviewed to compile this manuscript. The search was restricted to articles published in English language but no search filters with respect to time or area to keep the search results comprehenive.

## Case report

A 28-year-old female presented to the breast clinic with redness and mild pain in her right breast. On examination, the breasts were asymmetrical, a diffuse 15×10 cm lump was palpable in the upper inner quadrant of the right breast, with seropurulent discharge from multiple sinuses. Past medical history and the remainder of the physical examination were unremarkable. The discharge tested negative for acid-fast bacilli and tubercular antigen.

**Figure 1:**
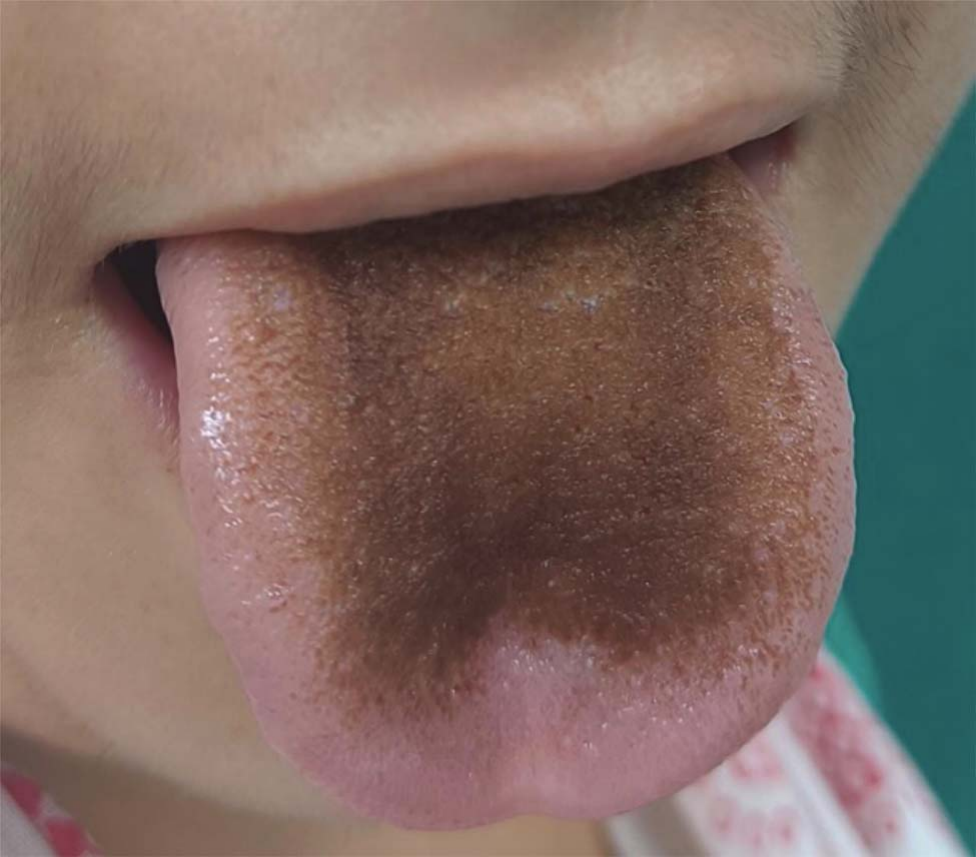
This image depicts discoloured hypertrophic elongated papillae on the dorsal surface of the tongue, characteristic of a linezolid-induced black hairy tongue.

**Figure 2:**
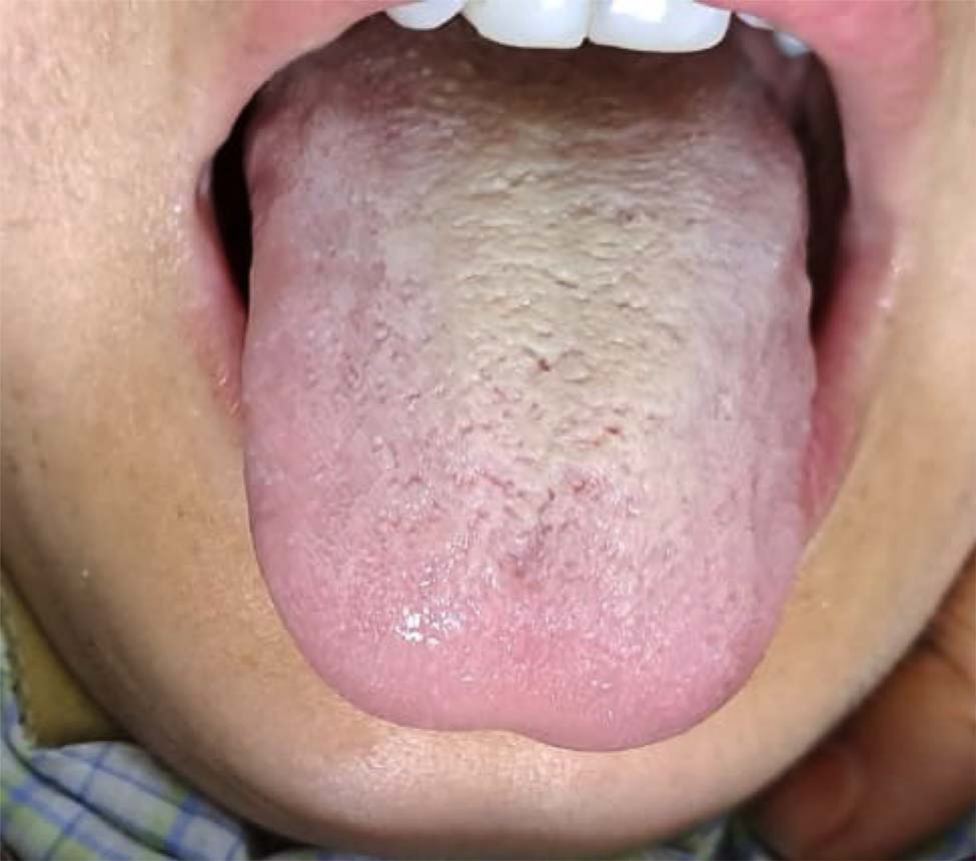
This image depicts the resolution of adverse effects 14 days following cessation of linezolid.

The patient received outpatient antibiotic management before evaluation, leading to negative cultures. A biopsy was done for histopathological examination, which confirmed the diagnosis of granulomatous mastitis. Based on antibiotic sensitivity and local resistance patterns, the patient was started on linezolid 600 mg OD and levofloxacin 750 mg OD. Acetaminophen and diclofenac were added for pain relief. The patient then followed up 5 days later with the complaint of blackish-brown discoloration of the tongue (Fig. 1). Linezolid-induced BHT was diagnosed and the drug was discontinued and the patient was advised to maintain good oral hygiene (rinse the mouth with water and alcoholic mouthwash twice daily). The tongue discoloration improved within 2 weeks of discontinuing linezolid as a dechallenge test (Fig. 2). The Naranjo Adverse Drug Reaction Probability Scale revealed a probable association (Tables 1,). The patient was started on a different regimen for Idiopathic Granulomatous Mastitis and improved on alternate drug therapy.

**Table 1:** Naranjo adverse drug reaction probability scale for the patient

Naranjo adverse drug reaction probability scale				
Question	Yes	No	Do not know	Score
1. Are there previous conclusive reports on this reaction?	+1	0	0	0
2. Did the adverse event appear after the suspected drug was administered?	+2	−1	0	+2
3. Did the adverse reaction improve when the drug was discontinued or a specific antagonist was administered?	+1	0	0	+1
4. Did the adverse event reappear when the drug was re-administered?	+2	−1	0	0
5. Are there alternative causes (other than the drug) that could on their own have caused the reaction?	−1	+2	0	0
6. Did the reaction reappear when a placebo was given?	−1	+1	0	+1
7. Was the drug detected in blood (or other fluids) in concentrations known to be toxic?	+1	0	0	0
8. Was the reaction more severe when the dose was increased or less severe when the dose was decreased?	+1	0	0	0
9. Did the patient have a similar reaction to the same or similar drugs in any previous exposure?	+1	0	0	0
10. Was the adverse event confirmed by any objective evidence?	+1	0	0	+1
Total score:	5			

ADR, Adverse Drug Reaction.

Here, Interpretation: doubtful ADR (<2), possible ADR (2–4), probable ADR (5–8), definite ADR (≥9)

## Discussion

BHT is a rare side effect of linezolid therapy. Also known as “lingua villosa nigra”, BHT is characterized by discoloured and hypertrophic elongated papillae on the dorsal surface of the tongue. The pathophysiologic mechanism for linezolid-induced BHT is not well understood. The hypothesized mechanism involves drug-induced defective tongue desquamation resulting in thickened and hypertrophied filiform papillae which, with debris and microbiological colonization lead to staining which in turn leads to BHT^4,6,9,10,14^. While aetiology remains unknown, some risk factors seem to aggravate BHT like smoking or chewing tobacco, alcohol consumption, black tea or coffee, drugs (like cocaine), poor oral hygiene, oxidative mouthwashes, cancer, neurological disorders (like trigeminal neuralgia, head or neck radiotherapy), acquired immune syndrome, drugs causing xerostomia, and certain antibiotics^4,6,14^.

The clinical diagnosis relies on visual observation and correlation with a detailed clinical history. Pathology evaluation may also be required on rare occasions. Treatment involves the identification and discontinuation of the offending agent. Other components of treatment include reassurance to the patient and maintenance of adequate oral hygiene. Gentle debridement has been used in select cases to promote desquamation^5^ The treatment recommendations made to our patient align with all prior case studies reported^4,6,9,10,12^.

The offending agent (linezolid) was instantly discontinued after the episode of BHT and the patient recovered in 2 weeks. The decision to discontinue the drug is better individualized in lines with the risk-benefit ratio for the patient. Interestingly, in a case study by Khasawneh *et al.* ^11^, the patient was recommended to continue the therapy despite the incidence of BHT. Similarly, in the case study by Lee *et al*.^6^, linezolid was discontinued but continued again despite the occurrence of BHT. The study also noticed that the degree of discoloration after reintroduction of linezolid was less severe with no related symptoms reported by the patient. Furthermore, Jain *et al*.^15^, showed that the reintroduction of linezolid therapy after an initial BHT incidence did not lead to the recurrence of BHT, and the drug was continued for 2 years without any such event.

In addition, our patient was reassured about the benign nature and good prognosis of the black tongue to alleviate the patient’s concerns and fear. The importance of patient counselling has always been highlighted in all such reports^5,6,11^. Table 2 presents a compilation of PubMed Indexed case reports and case series on BHT. On average, it takes about 2 weeks for the discoloration to appear after initiation of linezolid^14^. So, providers can also pre-warn the patients about when and what to expect after linezolid therapy initiation.

**Table 2:** Case reports and case series on linezolid-induced tongue discoloration and black hairy tongue indexed in PubMed database

Patient age	Sex	Indication for linezolid therapy	Concomitantly used medications	Naranjo score	Authors (first Name et al)	PMID	Ref
74	M	Coagulase-negative staphylococci infection	—	3	Dennis F Thompson *et al.*	20500047	^4^
60	F	MDR TB	Levofloxacin Amikacin Cycloserine Delamanid	8	Jaemin Lee *et al*.	34541939	^6^
80	M	Complicated right chronic pleural effusion	—	N/A	Cesare Braggio *et al*.	30333468	^9^
62	F	OM—foot	—	N/A	Ishma Aijazi *et al*.	25671958	^10^
56	M	CAP	Meropenem ertapenem	8	Faisal Abdullah Khasawneh *et al.*	23414605	^11^
5	M	Necrotic pneumonia with lung abscess of the right upper lobe	Meropenem Cefotaxime clindamycin	N/A	Theoni Petropoulou *et al*.	23934205	^12^
14	F	left orbital cellulitis	Meropenem Cefotaxime clindamycin	N/A	Theoni Petropoulou *et al*.	23934205	^12^
8	F	Bacteremia and Polyarthritis	Vancomycin	N/A	Jui-Shan *et al.*	19238109	^13^
14	M	MRSA pneumonia	Vancomycin	N/A	Shaohua Luo *et al*.	32944496	^14^
25	M	XDR TB	Capreomycin, Cycloserine, Moxifloxacin, Ethionamide and PAS	N/A	A K Jain	28166916	^15^
30	F	MDR TB	Kanamycin, Cycloserine, Ethionamide, Moxifloxacin, PAS	N/A	A K Jain	28166916	^15^
10	M	Post surgical infection of left side radial neck fracture	—	5	Govindan Balaji	25538341	^16^
42	M	Spondylodiscitis	—	N/A	Francisco Jover-Diaz	20634649	^17^
65	M	Multidrug-resistant Pseudomonas aeruginosa infection	Piperacillin–tazobactam	7	Jing Ren *et al*.	33044871	^18^

CAP, Community Acquired Pneumonia; F, female; M, male; MRSA, methicillin-resistant *Staphylococcus aureus*; MDR TB, Multi Drug Resistant; OM, Osteomyelitis; TB, Tuberculosis; N/A, not applicable; PAS, para-amino salicylic acid; XDR TB, Extensively drug-resistant Tuberculosis.

Lastly, we also support the recommendation of good oral hygiene^5,6,12^. The patient in our case was advised to rinse the mouth twice daily with water and mouthwash to maintain oral hygiene. Different reports have suggested varied means of achieving good oral hygiene. Most studies recommended gentle debridement of the tongue with a soft toothbrush and rinsing it off with baking soda or diluted hydrogen peroxide to help with desquamation^6,10,11^. Balaji and colleagues recommended cleaning the tongue with normal saline twice daily^16^. Whereas, Ren *et al*.^18^ recommended brushing teeth three times daily using a soft-bristle toothbrush and gargling after eating.

## Conclusion

This case report adds to the scarce literature on BHT secondary to linezolid and advocates for the promotion of oral hygiene when linezolid is used. We encourage patient reassurance when confronting a case of linezolid-induced BHT. The decision to discontinue the drug must be individualized weighing the risk- benefit ratio. We also recommend that when prescribing linezolid, providers should counsel patients on the potential side-effects (like BHT) and advise them to refrain from smoking and drinking black or green tea.

## Ethical approval

This case report has been reported in line with the SCARE guidelines.

## Patient consent

Written informed consent was obtained from the patient for publication of this case report and accompanying images. A copy of the written consent is available for review by the Editor-in-Chief of this journal on request.

## Source of funding

None.

## Author contribution

All the authors confirm the concept, design, data collection and analysis and interpretation, and writing are our own. We confirm there are no other contributors.Y.S. and I.P.: conceptualization, data curation, writing—original draft preparation, writing—reviewing and editing. M.F., O.K., and H.C.: Data curation, Writing—Original draft preparation, Writing—Reviewing and Editing. T.B.E.: conceptualization, writing—reviewing and editing, visualization.

## Conflicts of interest disclosure

None.

## Research registration unique identifying number (UIN)

Not applicable.

## Guarantor

Corresponding author and first author.

## Provenance and peer review

Not commissioned, externally peer-reviewed.

## Trial registry number

Not applicable.
